# Supplementary material for: nAdder: A scale-space approach for the 3D analysis of neuronal traces
Source: PLoS Comput Biol. 2022 Jul 5;18(7):e1010211. doi: 10.1371/journal.pcbi.1010211 (PMC9286273; doi:10.1371/journal.pcbi.1010211)
Supplement: S1 Text — Fig A: Different schemes for the intrinsic decomposition of 3D traces. (A) The green trace is entirely 3D. (B) The trace is decomposed by 1D line (pink) followed by 3D fragment (green). (C) The trace is decomposed by suite of 1D lines and 2D plane. (D) The trace is decomposed as sucsessive 2D planes. The decompositions are hierarchial as a 1D line is lying on a 2D plane, and the 2D plane itself is lying within a 3D portion. Fig B: Evaluation of intrinsic dimensional decomposition on simulated 3D traces. (A) Precision and Recall of the proposed nAdder algorithm and the baseline [37, 38] as a function of the noise level σ varying between 1 and 30 μm. The algorithms are applied at various scales from 1 to 100 μm and the scale with the largest accuracy (optimal scale) is chosen. (B) Comparison of estimated intrinsic dimensions at four different noise levels. Compared with the baseline approach, the nAdder is more robust to noise and gives much higher accuracies in both Precision and Recall. Details of the algorithms and simulations are shown in Methods. Fig C: Evaluation of the dimensionality decomposition algorithm at a fixed scale. Accuracy, Precision and Recall of the nAdder algorithm and the baseline approach from [37, 38] as a function of the noise level σ varying between 1 and 30 μm. The algorithms are applied at a fixed scale = 20 μm, which is small enough to avoid deforming the simulated curve. The accuracies of both algorithms are not as high as in the case of an optimal scale (Figure S1), but our approach still achieves ∼85% of accuracy at σ = 5 (medium noise) and ∼80% of accuracy at σ = 10 (high noise) for both 1D, 2D and 3D, compared to much lower accuracies for the baseline in 2D and 3D. Fig D: Intrinsic dimensional decomposition of the axonal trace of a retinal ganglion cell [40] across multiple scales. Positions on the 3D trace are indexed by the curvilinear distance u (x axis) and the scaled trace is calculated by Gaussian convolution with various standa [file pcbi.1010211.s001.pdf]

nAdder: A scale-space approach for the 3D analysis of neuronal traces

Minh Son Phan[1,2], Katherine Matho[3,4], Emmanuel Beaurepaire[1], Jean Livet[2], and Anatole Chessel*[1]

[1]Laboratory for Optics and Biosciences, CNRS, INSERM, Ecole Polytechnique, IP Paris, Palaiseau, France
[2]Institut Pasteur, Université de Paris Cité, Image Analysis Hub, F-75015 Paris, France
[3]Sorbonne Université, INSERM, CNRS, Institut de la Vision, 17 rue Moreau, F-75012 Paris, France
[4]Cold Spring Harbor Laboratory, Cold Spring Harbor, NY, USA
*anatole.chessel@polytechnique.edu

June 21, 2022

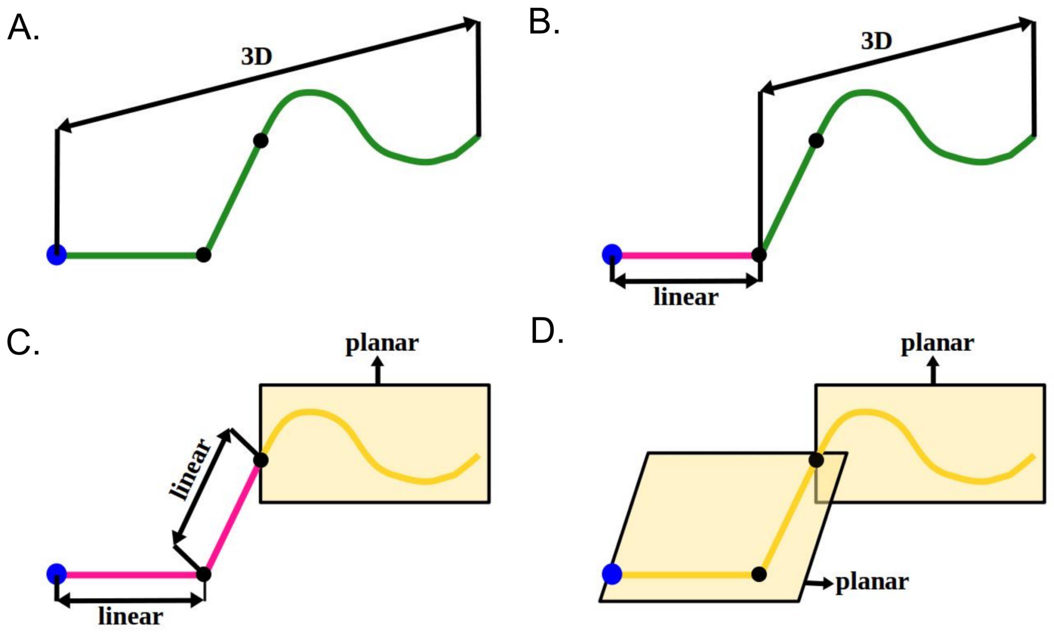

Fig A of S1 Text: Different schemes for the intrinsic decomposition of 3D traces. (A) The green trace is entirely 3D. (B) The trace is decomposed by 1D line (pink) followed by 3D fragment (green). (C) The trace is decomposed by suite of 1D lines and 2D plane. (D) The trace is decomposed as sucsessive 2D planes. The decompositions are hierarchial as a 1D line is lying on a 2D plane, and the 2D plane itself is lying within a 3D portion.

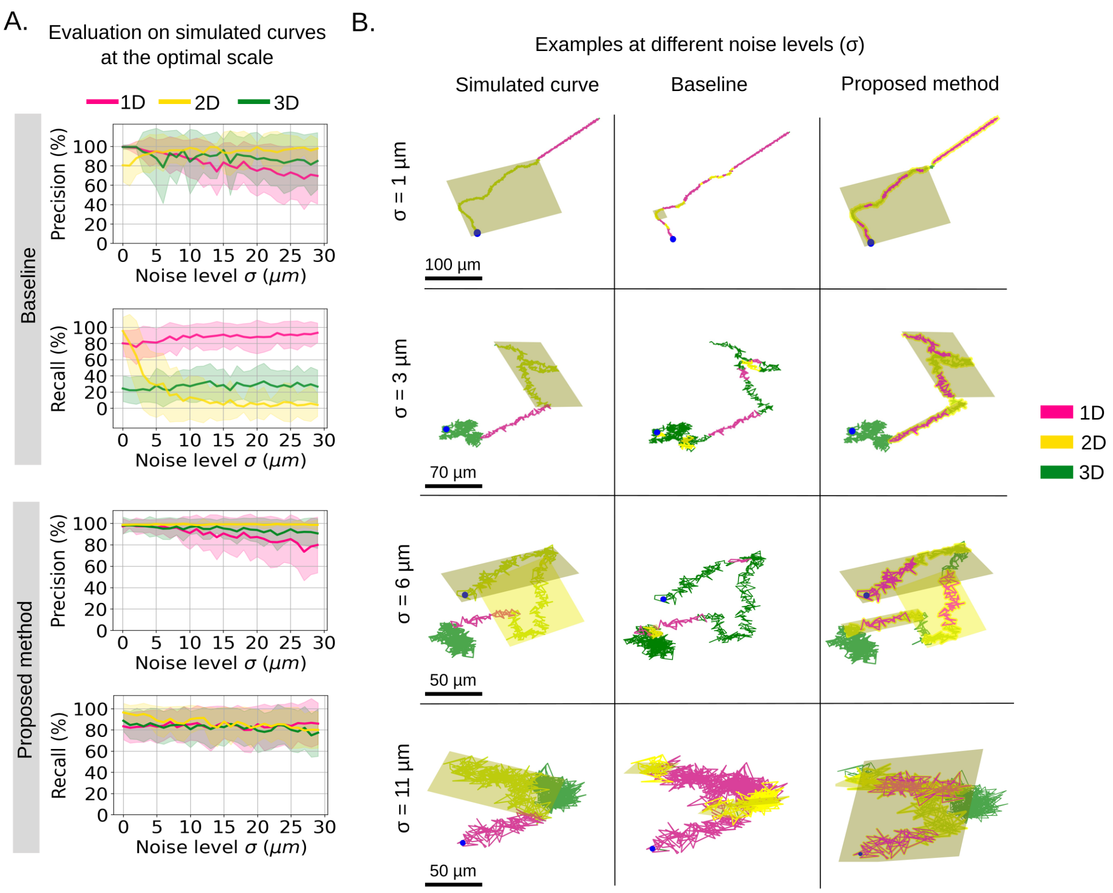

Fig B of S1 Text: Evaluation of intrinsic dimensional decomposition on simulated 3D traces. (A) Precision and Recall of the proposed nAdder algorithm and the baseline (1; 2) as a function of the noise level σ varying between 1 and 30 μm. The algorithms are applied at various scales from 1 to 100 μm and the scale with the largest accuracy (optimal scale) is chosen. (B) Comparison of estimated intrinsic dimensions at four different noise levels. Compared with the baseline approach, the nAdder is more robust to noise and gives much higher accuracies in both Precision and Recall. Details of the algorithms and simulations are shown in Methods.

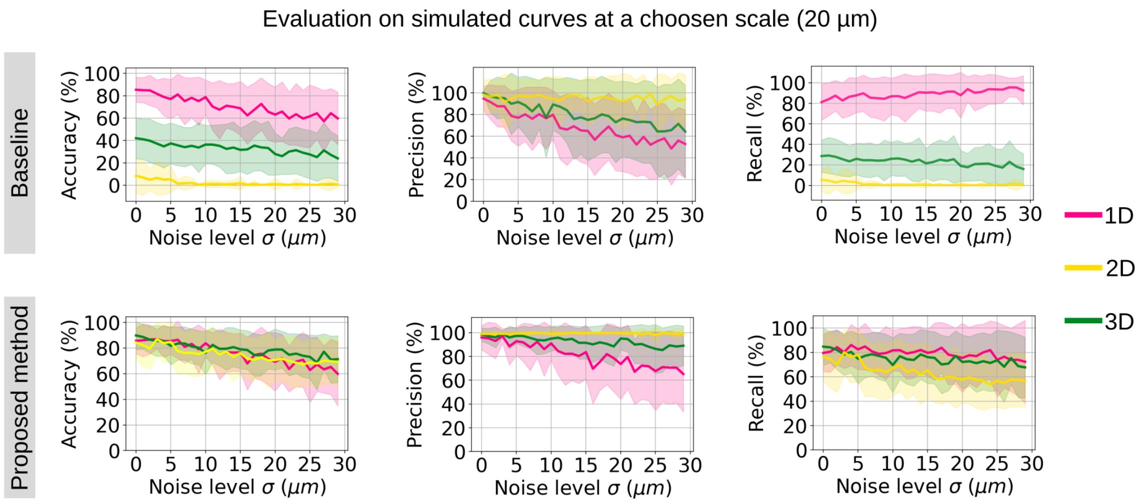

Fig C of S1 Text: Evaluation of the dimensionality decomposition algorithm at a fixed scale. Accuracy, Precision and Recall of the nAdder algorithm and the baseline approach from **(author?)** (1, 2) as a function of the noise level σ varying between 1 and 30 μm. The algorithms are applied at a fixed scale = 20 μm, which is small enough to avoid deforming the simulated curve. The accuracies of both algorithms are not as high as in the case of an optimal scale (Figure S1), but our approach still achieves $\sim$85% of accuracy at σ = 5 (medium noise) and $\sim$80% of accuracy at σ = 10 (high noise) for both 1D, 2D and 3D, compared to much lower accuracies for the baseline in 2D and 3D.

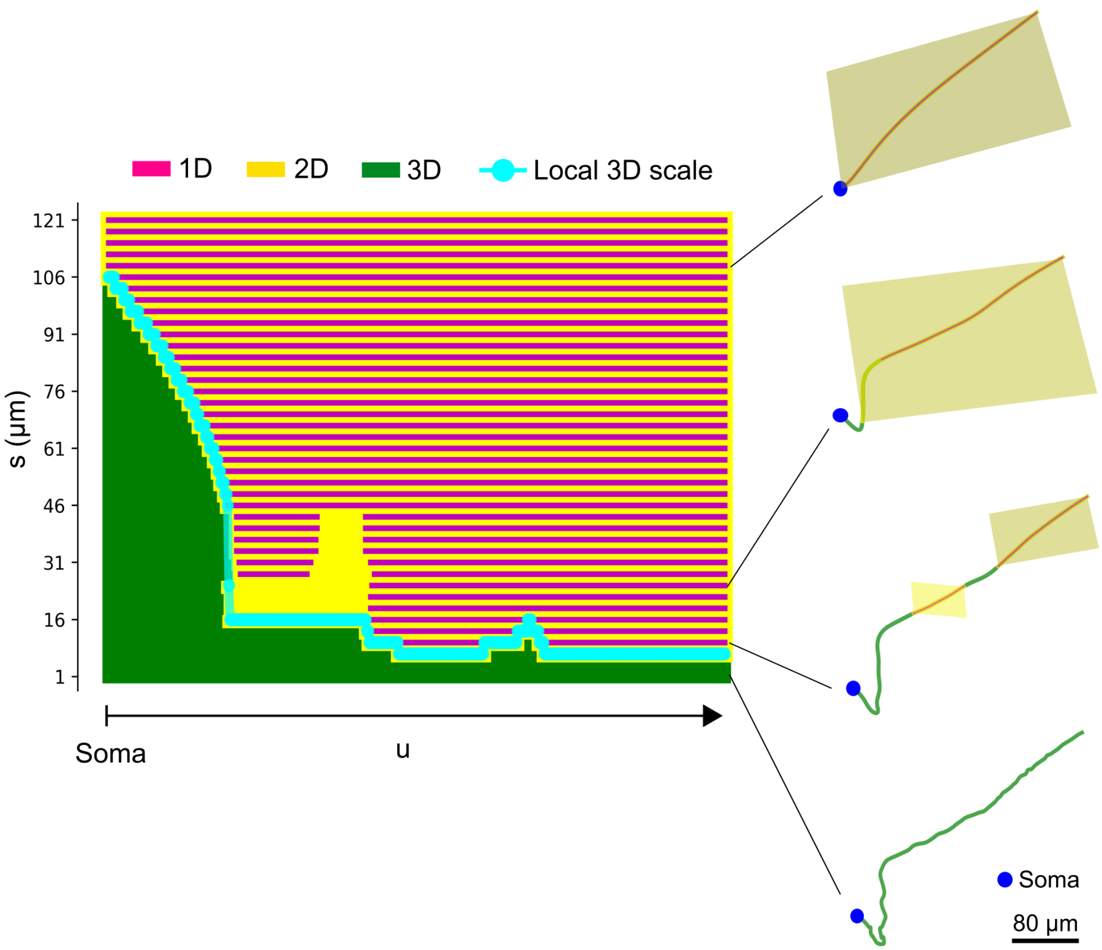

Fig D of S1 Text: Intrinsic dimensional decomposition of the axonal trace of a retinal ganglion cell (3) across multiple scales. Positions on the 3D trace are indexed by the curvilinear distance u (x axis) and the scaled trace is calculated by Gaussian convolution with various standard deviations s (y axis). An example of trace seen at different scales and superimposed with its decomposition is shown on the right. The trace exhibits mostly 3D at small scales, then decomposes into a combination of 1D/2D/3D portions at higher scales and finally transforms into a 1D line at a very high scale. The local 3D scale at each position u is then measured as the minimal scale s from that the dimension at u is not 3D any more.

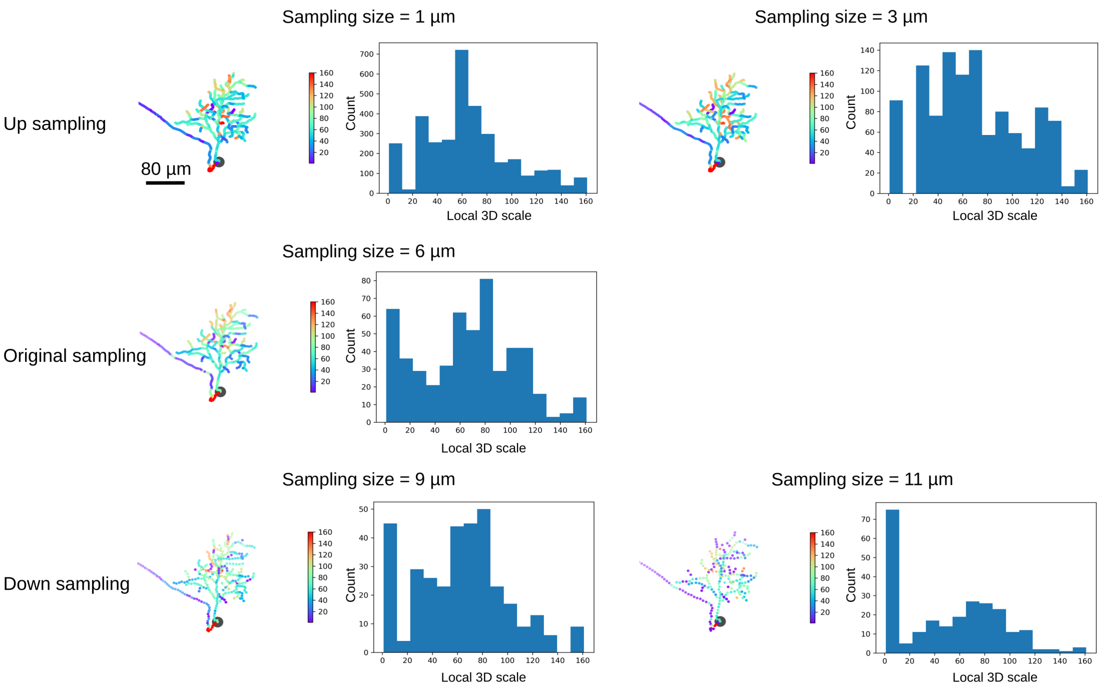

Fig E of S1 Text: Effect of sampling on local 3D scale computation Given a neuronal arbor (see Fig. 2 for details) whose original sampling is around 6µm, the local 3D scale was compued for several different value of over and under sampling. While we clearly see a difference, the overall behaviour is robust to changes in sampling rate.

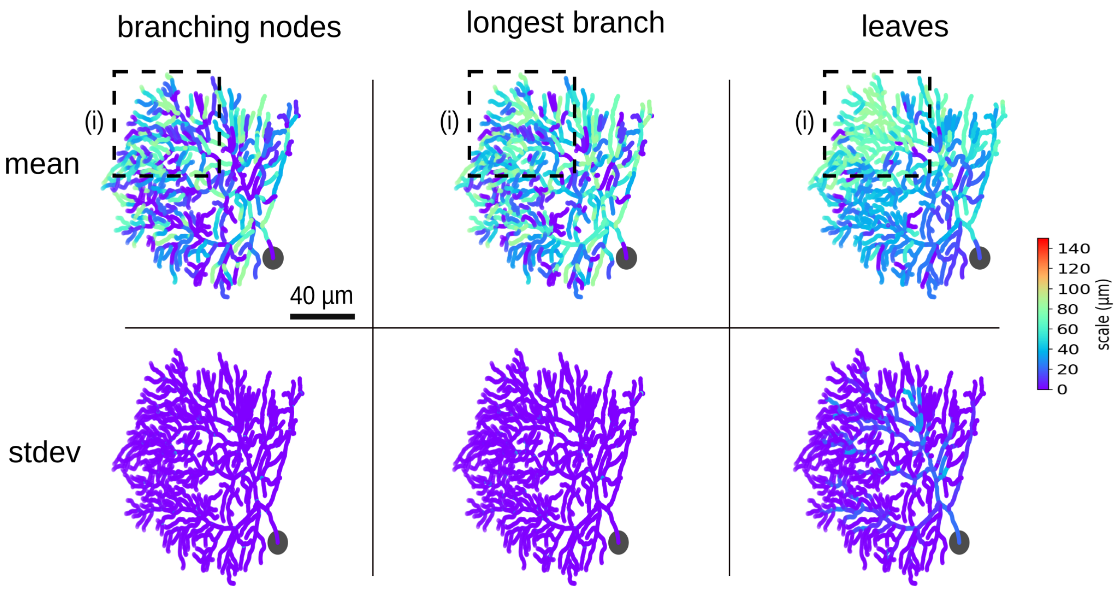

Fig F of S1 Text: Computation of local 3D scales in different decomposition modes on the cerebellar Purkinje neuron shown in Figure 2A3. (Left) neurite portions located between any two branching nodes were extracted. (Middle) the longest branch originating from the cell body (root tree) was first extracted, and the process repeated for all subtrees extracted from that longest branch. (Right) branches connecting the cell body to each dendrite termination ('leaf') were extracted. The mean and standard deviation of the local 3D scale was computed. The "leaves" mode (right) produces more stable local 3D scales with high and homogenous values in region (i) where the dendrites are sticking out of plane compared to the "branching nodes" (left) and "longest branch" modes (middle) (See S3 Movie).

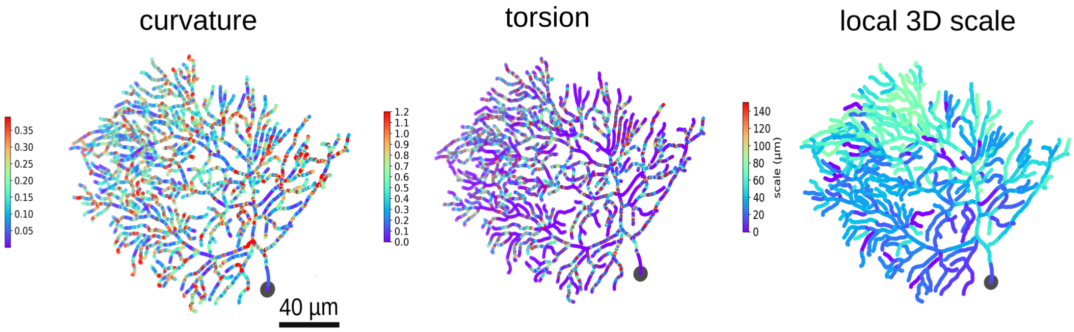

Fig G of S1 Text: Comparison between different local metrics of geometrical complexity. Three parameters, curvature (left), torsion (middle) and local 3D scale (right) were mapped on the cerebellar Purkinje neuron shown in Figure 2A3. The local 3D scale gives smoother values than those of curvature and torsion since it was computed using a scale space approach, and better contrasts different regions of the Purkinje cell's dendritic arbor.

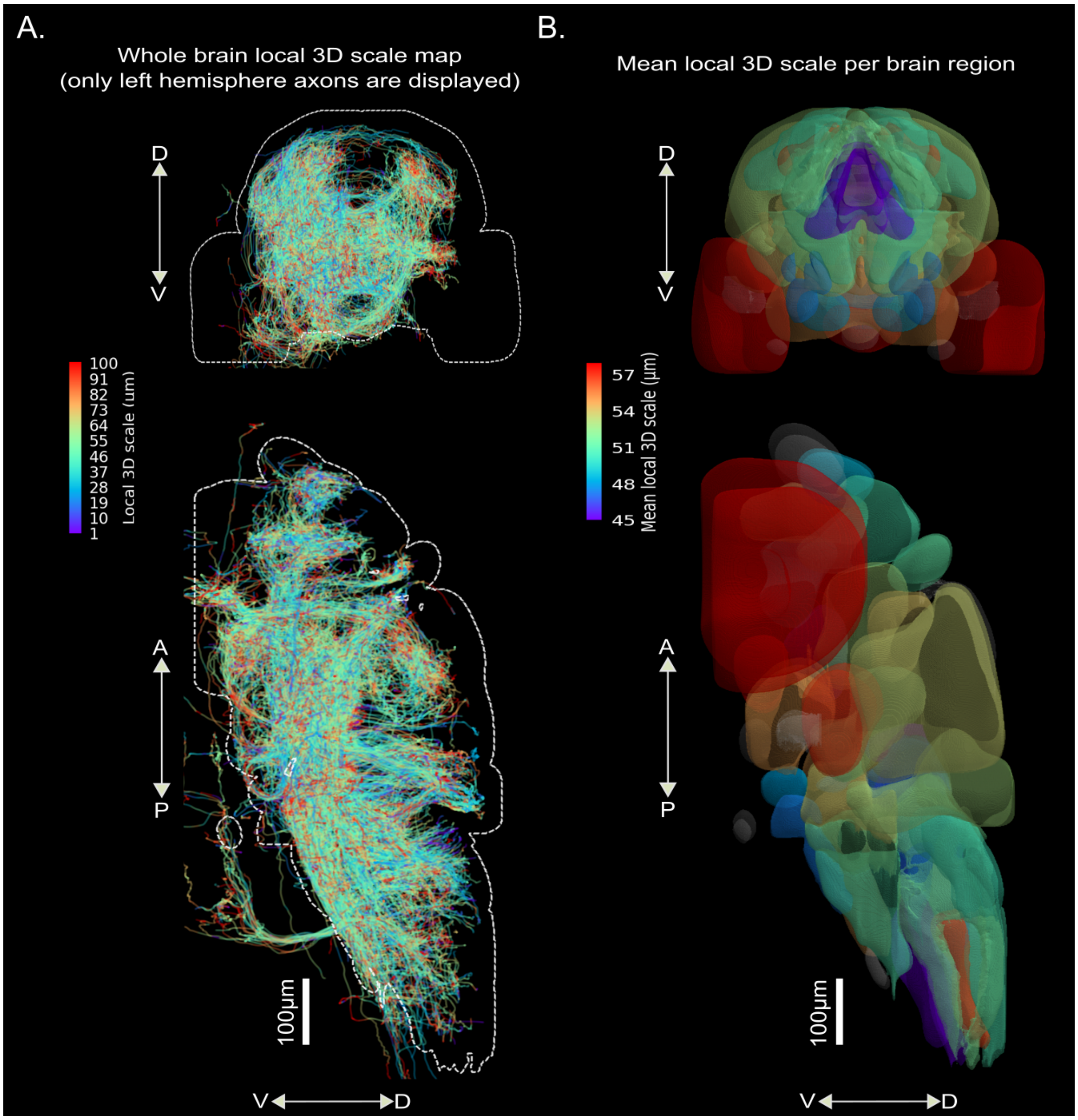

Fig H of S1 Text: Local 3D scales mapping across the whole larval zebrafish brain. The traces analyzed correspond to those presented in **(author?)** (4). (A) Coronal (top) and sagittal (bottom) views shiwing local 3D scale analysis of all axonal traces originating from the left hemisphere. (B) Mean local 3D scale by brain regions (values were clipped from 5th to 95th percentiles for clearer display). A transparency effect was applied to help visualizing inter-regional variations.

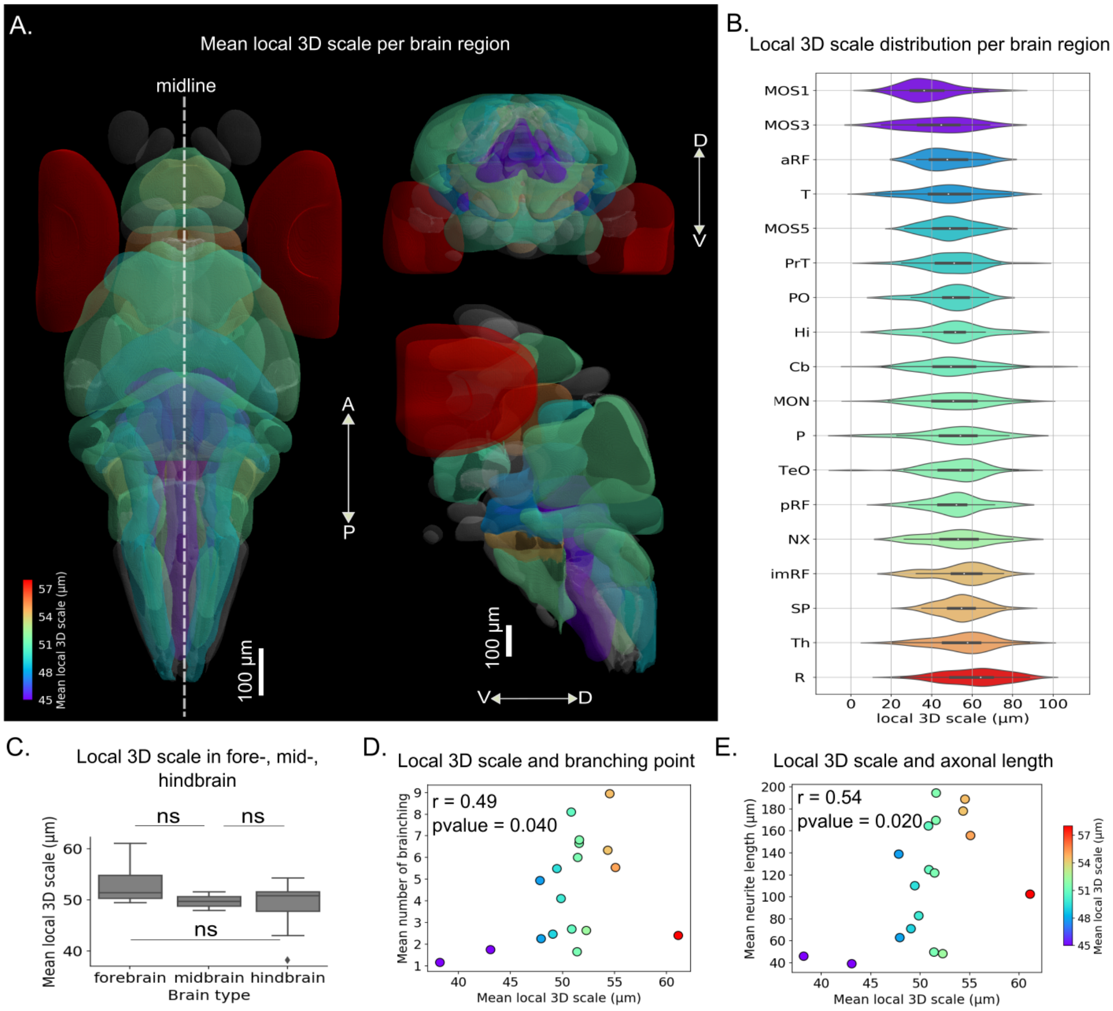

Fig I of S1 Text: Whole brain local 3D scale analysis of axons originating from different region of the larval zebrafish brain. Traces analyzed correspond to those presented in **(author?)** (4). (A) Mean local 3D scale by brain region (values were clipped in the same range as in Figure S6B for comparison). (B) Distribution of the local 3D scale values in each brain region. (C) Mean local 3D scale in fore- mid- and hindbrain. A Wilcoxon test with Holm-Sidak for multiple comparison was used, ns = not significant. (D, E) Correlation between the mean local 3D scales and average number of branching points (D) or trace length (E) in different brain regions. Spearman correlation was used.

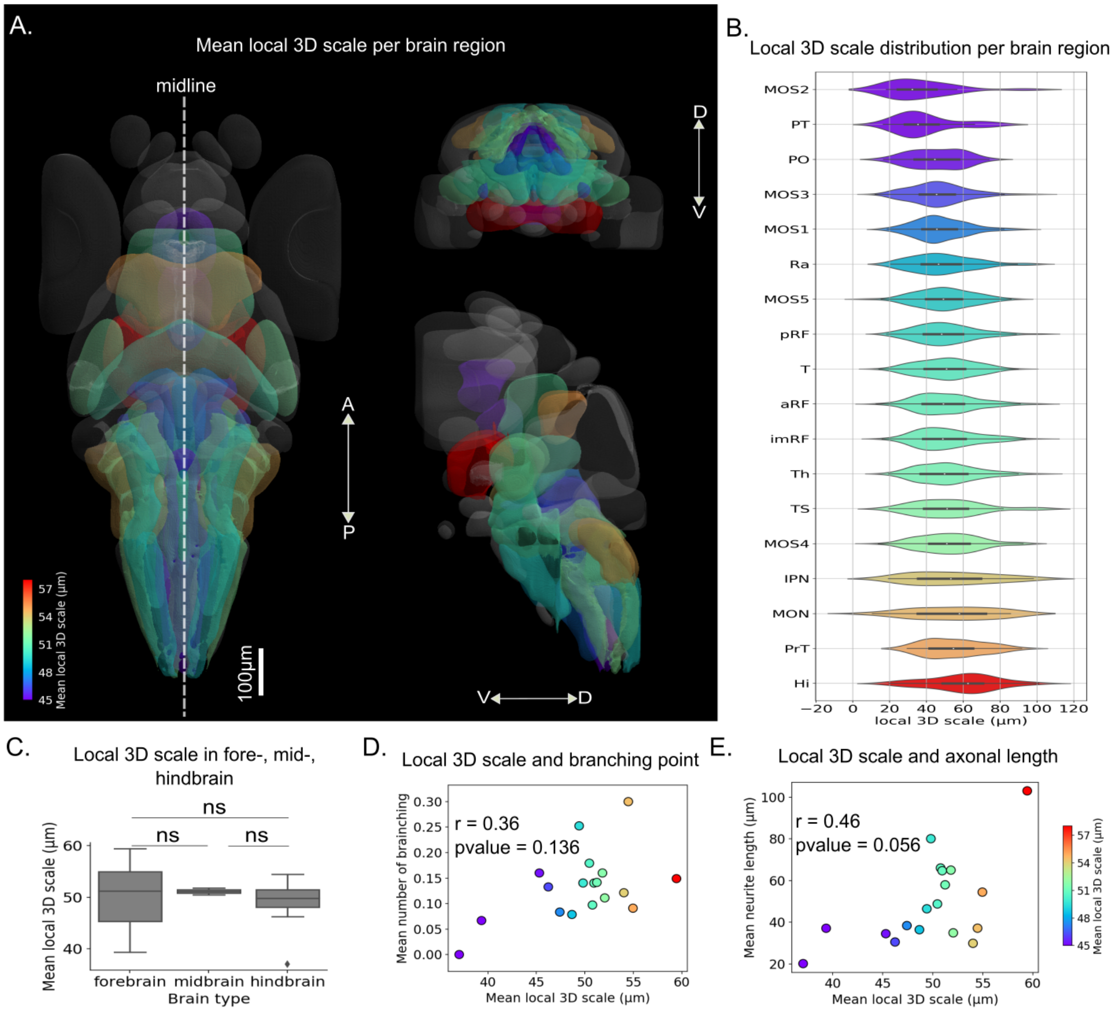

Fig J of S1 Text: Whole brain local 3D scale analysis of axons passing through different region of the larval zebrafish brain. Traces analyzed correspond to those presented in **(author?)** (4). (A) Mean local 3D scale by brain region (values were clipped in the same range as in Figure S6B for comparison). (B) Distribution of the local 3D scale values in each brain region. (C) Mean local 3D scale in fore- mid- and hindbrain. A Wilcoxon test with Holm-Sidak for multiple comparison was used, ns = not signficant. (D, E) Correlation between the mean local 3D scales and average number of branching points (D) or trace length (E) in different brain regions. Spearman correlation was used.

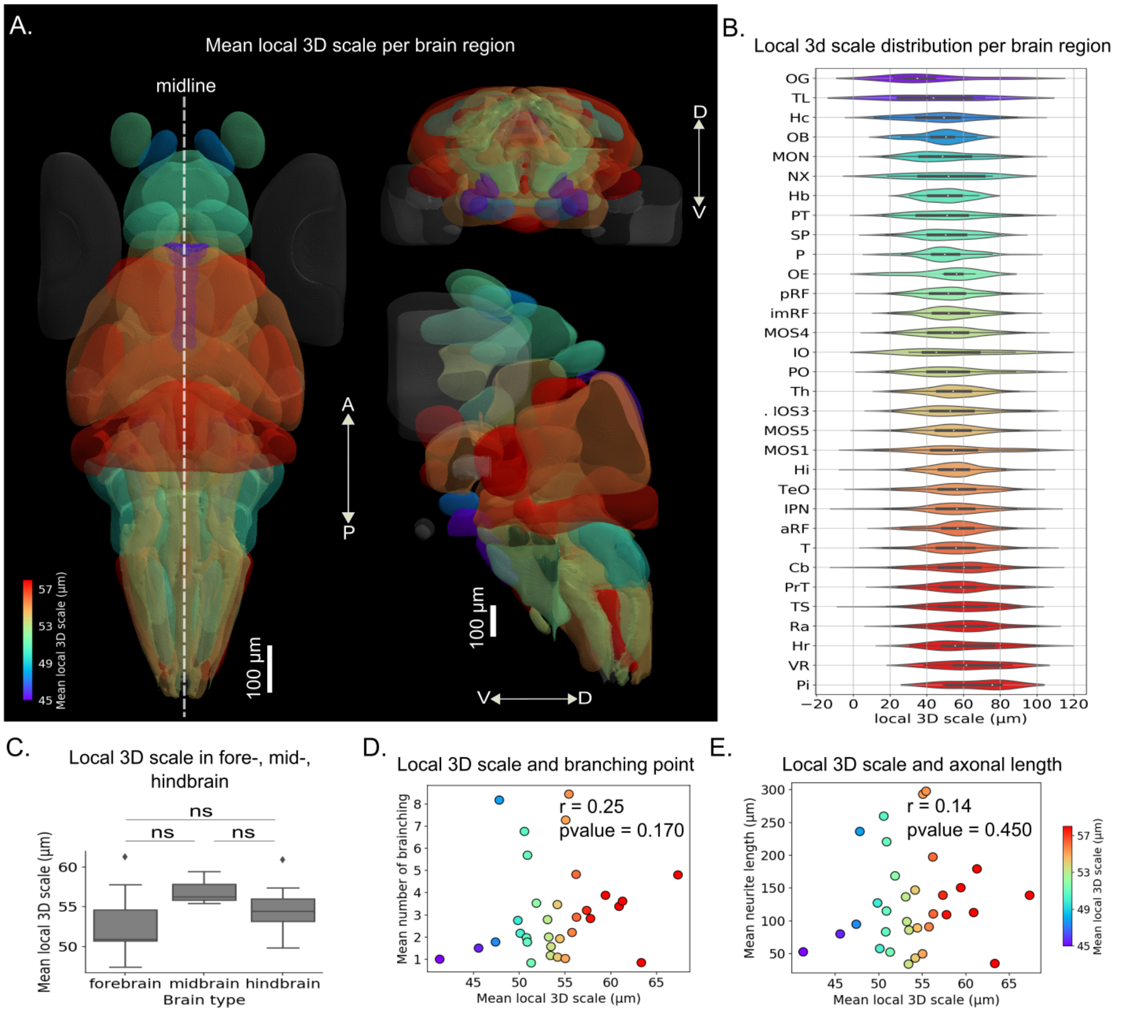

Fig K of S1 Text: Whole brain local 3D scale analysis of axons terminating in different region of the larval zebrafish brain. Traces analyzed correspond to those presented in (4). (A) Mean local 3D scale by brain region (values were clipped in the same range as in Figure S6B for comparison). (B) Distribution of the local 3D scale values in each brain region. (C) Mean local 3D scale in fore- mid- and hindbrain. A Wilcoxon test with Holm-Sidak for multiple comparison was used, ns = not signficant. (D, E) Correlation between the mean local 3D scales and average number of branching points (D) or trace length (E) in different brain regions. Spearman correlation was used.

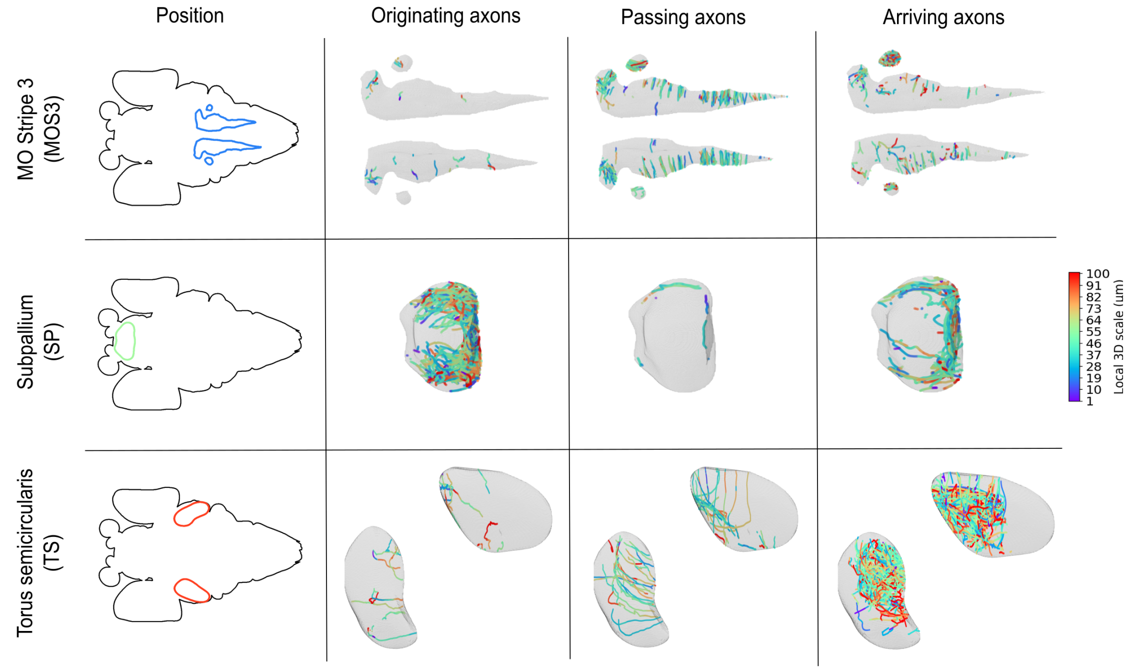

Fig L of S1 Text: Variability of local 3D scale for axons originating from, passing through or arriving in three brain regions (MOS3, SP, TS). The regional local 3D scale differs the three axons subset.

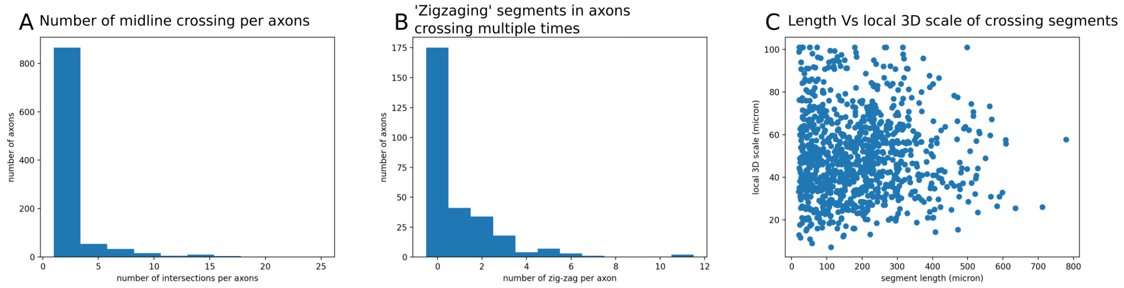

Fig M of S1 Text: Local 3D scale of axons crossing the midline. (A) Distribution of the number of midline crosses made by individual axon arbors. (B) Distribution of the number of 'zigzags' through the midline, i.e. total number of crossings minus the number of crossing branches, for axons crossing more than 2 times. (C) Relation between the local 3D scale and length of crossing branches at the midline. A branch is defined as a neurite segment located between two branching points, or a branching point and a leaf or the root of the arbor.

Supp. Algorithm A

Local 3D scale computation.

Supp. Algorithm 1: Local 3D Scale.

/* Let γ be the current curve, suitably sampled, γ_s the curve convolved with a gaussian kernel $\mathcal{N}(0, s)$, and r_{step} and r_{max} the maximum and interval of radius values investigated. */

foreach $r_{cur} \in [0, r_{step}, \ldots r_{max}]$ **do**
  $S_{r_{cur}}$ = ComputeScaleSetFromRadius(r_{cur},γ)
  $D_T(r_{cur})$ = Compute3DIndicatorFromScales($S_{r_{cur}}$,γ)
foreach $u \in [0, 1]$ **do**
  l3Ds(u) = min (r s.t. $\exists w \in D_T(r)$ s.t. $u \in w$)

Function `ComputeScaleSetFromRadius(`r,γ`):`
  S = []
  compute κ_s the curvature of γ_s for all s.
  foreach $u \in [0, 1]$ **do**
    S.append(min(s) s.t. $\frac{1}{\kappa_s(u)} > r$)
  return S

Function `Compute3DIndicatorFromScales(`S,γ`):`
  s_μ = mean(S)
  δs = stddev(S)
  foreach $s \in [s_\mu - \delta s, s_\mu + \delta s]$ **do**
    /* Compute curvature κ_s and torsion τ_s */
    Compute γ_s, then compute the 1st, 2nd and 3rd derivatives of γ_s, finally compute κ_s and τ_s.
    /* Compute T_s the 3D indicator */
    foreach $u \in [0, 1]$ **do**
      $T_s(u) = 0$
      if $\kappa_s(u) \leq \varepsilon_\kappa$ *or* $|\tau_s(u)| \leq \varepsilon_\tau$ **then** $T_s(u) = 1$
    Compute $\mathcal{D}_{T_s} = \{\omega_{s,k}\}$ lists of n_s distinct 1-labels intervals of T_s, for $k \in [\![1, n_s]\!]$
  Compute D_T by selecting from $\mathcal{D}_{T_s}{}_{s \in S}$ intervals that are stable across S, see text and implementation for details
  return D_T

Table A of S1 Text: Summary of 36 annotated brain regions from (author?) (4).

Index	Name	Abbr	Total_Nbaxon	Nbaxon_Orig	Nbaxon_Pass	Nbaxon_Arr	Mean_Local_3D_Scale
1	Medial_octavolateral_nucleus	MON	281	115	20	146	50,81
2	Cerebellum	Cb	365	224	10	131	53,48
3	MO_stripe_1	MOS1	355	181	108	66	44,14
4	MO_stripe_2	MOS2	28	7	19	2	39,26
5	MO_stripe_3	MOS3	386	16	294	76	47,66
6	MO_stripe_4	MOS4	201	13	108	80	52,31
7	MO_stripe_5	MOS5	278	73	103	102	51,16
8	interpeduncular_nucleus	IPN	150	0	33	117	55,38
9	inferior_olive	IO	13	0	7	6	61,06
10	caudal_hypothalamus	Hc	70	2	10	58	48,56
11	Raphe_nucleus	Ra	332	8	140	184	55,28
12	Tegmentum	T	505	71	173	261	53,1
13	anterior_reticular_formation	aRF	727	16	216	495	54,41
14	intermediate_reticular_formation	imRF	581	18	214	349	52,33
15	posterior_reticular_formation	pRF	562	37	171	354	51,23
16	Glossopharyngeal_ganglion	GG	2	1	0	1	66,08
17	Habenula	Hb	34	6	3	25	51,71
18	intermediate_hypothalamus	Hi	366	21	47	298	55,38
19	rostral_hypothalamus	Hr	31	2	3	26	59,67
20	Octaval_ganglion	OG	19	3	10	6	47,8
21	Olfactory_bulb	OB	41	9	3	29	49,1
22	Olfactory_epithelium	OE	14	7	1	6	48,41
23	Pallium	P	110	18	6	86	51,13
24	Pituitary	Pi	5	0	0	5	67,33
25	Posterior_teberculum	PT	76	12	30	34	46,71
26	preoptic_region	PO	127	19	50	58	49,71
27	pretectum	PrT	231	68	55	108	54,65
28	Retina	R	51	47	1	3	60,71
29	subpallium	SP	105	67	3	35	52,94
30	tectum	TeO	195	57	2	136	54,2
31	Thalamus	Th	437	67	106	264	53,59
32	Torus_longitudinalis	TL	13	8	1	4	46,27
33	Torus_semicircularis	TS	195	11	50	134	57,53
34	Trigeminal_ganglion	TG	4	3	1	0	57,1
35	Vagal_region	VR	31	10	8	13	57
36	vagus_motor_neurons	NX	46	30	4	12	53,18

Table B of S1 Text: List of regions having axons originating from and arriving to the Torus Semicircularis (TS) from (author?) (4). Index 0 corresponds to axons not starting from any regions.

Index	Name	Abbr	Number
0	NaN	NaN	43
1	Medial_octavolateral_nucleus	MON	21
2	Cerebellum	Cb	4
5	MO_stripe_3	MOS3	3
6	MO_stripe_4	MOS4	2
7	MO_stripe_5	MOS5	11
12	Tegmentum	T	6
13	anterior_reticular_formation	aRF	2
14	intermediate_reticular_formation	imRF	3
18	intermediate_hypothalamus	Hi	2
19	rostral_hypothalamus	Hr	1
25	Posterior_teberculum	PT	1
27	pretectum	PrT	5
30	tectum	TeO	28
31	Thalamus	Th	7
35	Vagal_region	VR	2

References

[1] Yang J, Yuan J, Li Y. Parsing 3D motion trajectory for gesture recognition. Journal of Visual Communication and Image Representation. 2016;38:627–640. doi:10.1016/j.jvcir.2016.04.010.

[2] Ma X, Zhao T, Wen R, Wu Z, Wang Q. Motion recognition based on concept learning. In: I2MTC; 2017. p. 1–6.

[3] Badea TC, Nathans J. Morphologies of mouse retinal ganglion cells expressing transcription factors Brn3a, Brn3b, and Brn3c: analysis of wild type and mutant cells using genetically-directed sparse labeling. Vision Research. 2011;51(2):269–279. doi:10.1016/j.visres.2010.08.039.

[4] Kunst M, Laurell E, Mokayes N, Kramer A, Kubo F, Fernandes AM, et al. A Cellular-Resolution Atlas of the Larval Zebrafish Brain. Neuron. 2019;103(1):21–38.e5. doi:10.1016/j.neuron.2019.04.034.
