# Supplementary material for: nAdder: A scale-space approach for the 3D analysis of neuronal traces
Source: PLoS Comput Biol. 2022 Jul 5;18(7):e1010211. doi: 10.1371/journal.pcbi.1010211 (PMC9286273; doi:10.1371/journal.pcbi.1010211)
Supplement: Attachment — Submitted filename: ResponsesToReviewers.pdf [file pcbi.1010211.s005.pdf]

Authors Responses to Reviewers Comments

Paper title: nAdder: A scale-space approach for the 3D analysis of neuronal traces
Minh Son Phan, Katherine Matho, Emmanuel Beaurepaire, Jean Livet, and Anatole Chessel

The authors would like to thanks the editors and reviewers for taking the time to carefully read and comment on our work. You will find a description of the changes we made and the responses to your comments below.

Reviewer #1: # General

Disclaimer: I am neither an expert in geometry nor in neuro-science, and I may thus overlook aspects that experts in those fields may spot.

For my point of view (possibly representing a general reader with a background in biophysics) I would like to congratulate the authors for this very well written manuscript! The presented method strikes me the same time as understandable, feasible and biologically meaningful! I'd anticipate a broad adoption of the method in the field of neuron trace inspection and classification.

I only have a few suggestions for improvements of the manuscript, figures, and software documentation.

We thank the reviewer for his/her positive evaluation of our method and manuscript.

Manuscript text

Line 8: Could you add the gitlab link to the software to the abstract to facilitate uptake of the software?!

The link is now in the abstract.

Line 106: I know one could look this up in the original publication, but would it be possible to add two sentences explaining how the convolution of a trace works? I assume one loops through the coordinates and replaces each coordinate with a weighted mean of the coordinates in the vicinity?!

A few words of explanation have been added.

Line 284: You speak about "fragments", could you please add an explanation to the manuscript what a "fragment" is? Is it something like "branches" (a term used in skeletonization of images)?

Here a fragment is a piece of curve with a constant dimensionality. A few words of explanation have been added here as well.

Line 352: "...as a new local metric...". Could you please add some discussion to the manuscript (maybe in the methods section) as to "how local" this really is? Since you need up to the 3rd derivative to compute the scale I guess you need at least some neighboring points on the trace? How much distance does this correspond to in micrometer units? Does this depend on the sampling of the trace? Related to that, do the traces need to be sampled equidistant for the method to work?

"How local" the measurement is linked to the scale at which the 3D structure of the curve disappears, which is linked to the size of the gaussian kernel used to smooth it.At the scale used to compute the local 3D scale, all structures smaller (i.e. with a smaller radius of curvature) than that computed local 3D scale has been smoothed out; so, the measurement is made at a size of the order of magnitude of the local 3D scale itself.

Some of those points are answered more fully in the answers to Reviewer 2 below; in particular we included supplementary figure S5 studying the effect of sampling.

Manuscript figures

Legend for 1D,2D,3D

Could you please make the coloring coding for 1D,2D,3D consistent throughout the manuscript.
- Fig 1A has colors with dashes
- Fig 1B middle panel, is really hard to see (at the small print size), are there some yellow boundaries around 1D? Could they be removed to make it easier to look at?
- Fig S2 simple colors
- Fig S3 same as 1A
- Fig S4 there is some checkerboard pattern for 1D

I'd recommend a simple color-blind safe scheme without any borders or dashes or alike, e.g. the scheme in Fig S2 would work for me.

The legend has been amended

Scale bars

Please add scale bars such that one can see how large the trace is. Scale bars are missing in Figures: 1B, S2, S5, S6

The missing scale bars have been added.

Software

Notebook link broken

This notebook can be download from:
https://gitlab.com/genepy3d/genepy3d/-/blob/master/docs/source/working_with_curve.ipynb

Yields: "docs/source/working_with_curve.ipynb" did not exist on "master"

The link has been changed.

Reviewer #2: The authors are introducing a novel local descriptor for space-curves. They are computing this new descriptor in existing datasets of neuronal arbors, and show that it is complementary to previously used morphological measurements of neuronal traces. Overall, the authors show that you can use this 3D scale metric for a fine neuronal trace description, which can bring more insights when comparing experimental conditions. This could be useful for a variety of neuroscience fields, and the applied data looks convincing.

However, after going through the paper several times, I still can't get a clear understanding of what this local indicator is measuring. I don't know how to interpret it and I wonder how the audience of neuroscientists will be willing to use a descriptor which looks quite complicated to understand.

I explain below a few points which mislead me and which could also mislead readers. I hope that my misdirections can be used by the authors to rewrite some parts of the paper to make it more easily understandable.

We thank the reviewer for his/her careful reading of the manuscript and comments and suggestions that hopefully enabled us to make the method section more understandable.

1 - Intuitively, when looking at a curve at a small scale, a 1D description is sufficient, because the curve follows its tangent. Then, looking a bit further away (higher up in the scales), one could use a plane (2D) to account for the curvature. Finally, at a higher scale, torsion has to be taken into account: the 3rd dimension becomes necessary to describe the curve. Following my wrong 'intuition', I was expecting that a 3D description is required at high scales, and a 1D description would be sufficient for low scales. But this intuition is wrong because Fig. 1B gives the inverse relation (small scale (15): 3D, large scale (150): 1D).

What I did not initially get is that the scale shown in the Y axis of Fig1B represents the degree of smoothing of the curve. Thus when the curve is heavily smoothed (150 microns), it can be 'modeled' as a line. Conversely, when it is less smoothed, it needs to be described within a 3D space. Ok.

However I do not really understand how you can derive a local property out of this observation. To get a local property, I believe that a FIXED 'typical sampling size' (let's say 5 microns for instance) has to be introduced, independent from the smoothing scale. This sampling size would be the one to

which you are comparing your smoothed curve at a particular scale. If no ‘typical sampling size’ is introduced, then looking with a 50 micron precision at a 50 micron-smoothed curve will give more or less the same ‘dimensionality’ as looking at a 10-micron smoothed curve with a 10 micron resolution.

So: shouldn’t there be an independent sampling size parameter for which the curve is analyzed? If there is an implicit one, where does it come from?

Indeed, sampling is an important parameter that we should have discussed. It can be seen as an additional way through which a scale space of a curve can be constructed, with great subsampling leading to a simplified curve and as such is best kept constant so as to not interfere with the nAdder algorithm. Indeed, in all the experiments shown in the paper, we kept it constant at 1 µm. One way to set this parameter is related to the Shannon-Nyquist sampling theory: it should be small enough compared to the resolution of the underlying imaging/tracing method and/or the expected size of the smallest detail in the trace that no information is lost via sampling.

The new Fig. S5, investigating the effect of sampling, show that the measured local 3D scale is fairly robust to changes in sampling. We added the new supplemental figure and a discussion on that topic in the methods sections 3.3.

2 - In the abstract:
“we propose a local tridimensional (3D) scale metric derived from differential geometry, defined as the distance in micrometers along which a curve is fully 3D as opposed to being embedded in a 2D plane or 1D line”.

What’s surprising is that ‘locally’ a differentiable curve is a line, thus 1D. If you sample the curve over a finite portion, at a certain size, then I understand that you may consider the curve being 1D or 2D or 3D over this portion. But there is no mention of a ‘typical size’ in the abstract, thus leading to my confusion. Is there any way this sentence can be rewritten to understand better the distance over which the curve is considered 3D?

That sentence was indeed misleading. The new abstract reads:

“we propose a local tridimensional (3D) scale metric derived from differential geometry, measuring for each point of a curve the characteristic length where it is fully 3D as opposed to being embedded in a 2D plane or 1D line. The larger this metric is and the more complex the local 3D loops and turns of the curve are.”

Which make clearer the interpretation of the proposed measurement and gives an intuition into it’s locality.

3 - In materials and methods:
I think part of the answers to my questions about the ‘typical size’ is answered in L270 to L283. But here I do not really understand the logic for the complicated scaling. Why not always choose a fixed set of scales with a standard set of sizes (2 microns, 4 microns, 8 microns, up to infinity)? Wouldn’t

that be easier to understand ? Would that impact the results ?

That explanation is indeed a bit confusing. The underlying algorithm add some internal complexity, with the aim of keeping external simplicity, i.e. to have parameters that are interpretable and easier to set.

A scale-space is used twice in nAdder. First to compute the dimensional decomposition robustly around a given scale, then in the computation of the local 3D scale proper to look at dimensional decompositions across scales. In the second case, we indeed do go through a list of standard scales, from 0 to a max scale that depends on the dataset, as shown in the graphical example of that computation in Fig S4.

In the first case, to look around a given scale of interest, we implemented the more complex scheme described L270 to L283. Its aim is two folds: 1) to define the scale of interest in micron based on the actual level of detail of the curve at that scale (the radius of curvature of the smallest detail left at that scale), 2) to obtain for each curve a list of scales, around that scale of interest, to go through to compute the hierarchical decomposition.

Again the point of this added complexity is to have fewer and simpler free parameters.

The methods were amended to be clearer on those points.

Overall, I believe that a longer and broader method section that precisely goes through all steps of the math is required to make the metric easier to understand. Many synthetic simple example curves throughout this section could lead to a better understanding of the method. (broken lines, spirals, lines intertwined with circles, etc.).

There is one example in supp Fig.1, but I do not know how the scale decomposition shown in Fig1B would look like with this the supp Fig. 1 example. Also, how the supp Fig S4 would look if color coded like the Fig 1B (right) ?

Fig S4 have been modified to keep the same color code as Fig1 and the rest of the figure. It has also been amended to show graphically approximately how the local 3d scale is computed from the scale space.

A more exact and exhaustive pseudo-code description of the algorithm has been added to the supplements.

Adding a synthetic example could be misleading as they are special cases that are not encountered in practice and where the behavior of the algorithm would not reflect how it works in general. While not dwelling into details, Fig 2A shows how the algorithm works by displaying the decomposition of several actual arbors at different scale as well as the final local 3D scale.

We hope that together, the changes we made and the supplemental material we added are sufficient to address reviewer 2’s concerns and make the methods sections mode understandable.

Some other remarks:

It is unclear to me what the graphs in figure 1A (right) are representing, and the caption is not helpful.

The caption has been improved to clarify that point.

L274 “For a given curve, the level of detail can be characterized by the maximal rκ along that curve.” -> unclear which level of detail ? I guess the lowest level of detail. But isn’t the lowest level of detail just a line joining both ends (with an infinite rk) ?

It would refer to the level of details kept at that scale from that curve. The text has been amended,

Typos:

L263 diferentiable -> differentiable
L276 keeing -> keeping

Corrected.

Reviewer #3: The authors provide a novel metric to parametrize a 3D curve. They call it Neurite Analysis through Dimensional Decomposition in Elementary Regions or ‘nAdder’. The metric is based on estimating the local 3D scale along a curve.
The concept of the proposed metric is well presented in Fig 1. Then, the authors highlight the usefulness of the proposed metric to characterize differences in previously traced neurons where traditional metrics failed to revealed any significant difference (Fig. 2B). To showcase the ability of the new metric to generate insights within larger datasets authors use two examples, the whole larval zebrafish brain (Fig. 3) and the axonal behavior along defined anatomical locations and along specific tracts. Both examples show clearly the utility of the proposed metric in characterizing neuronal populations and brain regions.

Overall, the paper in its current form is well-written, clear, with carefully selected examples where the new metric generates new insights and is well-discussed. I see no need to add any further comments or request additional material.

I would love to see in the future how this new metric can be paired with molecular profiling experiments to compare geometric phenotype with the proteomic or transcriptomic profiling of individual neurons.

We thank the reviewer for this positive assessment of our work.

A single minor suggestion, to the sentence of p4 line 112: “we define the local 3D scale as the highest scale at which the trace still remains 3D” somehow the concept that is a local measure is not included. Could this be rephrased to include that information?

A clearer formulation could be “we define the *local 3D scale* at a position as the highest scale at which the trace still remains locally 3D around that position”. We amended the text. See also the clarifications made as answers to reviewer 2’s comments.
